# From Testicle to Brain: A Case of Disseminated Tuberculosis

**DOI:** 10.7759/cureus.38526

**Published:** 2023-05-04

**Authors:** Mariana Fidalgo, Joana Cabral, Inês Soares, Marta Oliveira

**Affiliations:** 1 Internal Medicine, Centro Hospitalar Vila Nova Gaia/Espinho, Vila Nova Gaia, PRT; 2 Internal Medicine, Centro Hospitalar Vila Nova Gaia Espinho, Vila Nova Gaia, PRT

**Keywords:** public health, medical error, tuberculous meningitis, disseminated tuberculosis, tuberculosis

## Abstract

Tuberculosis remains a major cause of death by infection in the world. Disseminated tuberculosis occurs most frequently in the context of reactivation of a previously latent infection and is invariably lethal if untreated. Age, late presentation, and serious underlying disease are strong death predictors. We report the case of a 72-year-old male patient who presented to the emergency room with sudden onset hemiparesis and aphasia, with no acute lesions on contrast CT. Two months prior to the current event, the patient had undergone surgery for a testicular abscess in a different hospital. Since the surgery, he had progressive and unexplained weight loss and dysphagia. The medical team reviewed patient records from this hospital and the one where the surgery took place and concluded that the histopathology results from the surgery were not reviewed in the post-surgery follow-up consult and that the diagnosis of genitourinary tuberculosis was never made. This disease, untreated, evolved into disseminated tuberculosis with central nervous system involvement, causing the neurological deficits the patient presented and leading to his death. Surveillance and notification systems exist for individual and public health safeguarding. In the present case, failure to review the pathology report after surgery, coupled with the absence of notification from the laboratory, delayed the diagnosis and led to patient death. This report suggests a need for continuous system improvement, with integrated healthcare records and interinstitutional communication channels, in order to minimize information loss, diagnostic delays, and public health risks.

## Introduction

In the XXIst century, tuberculosis (TB) remains a leading infectious cause of death worldwide, with the human host being one of the main reservoirs for *Mycobacterium tuberculosis* [1,2]. Disseminated tuberculosis occurs when the microorganisms spread from the primary site of infection (the lungs) to other parts of the body through the blood or lymphatic system. Immunocompromised hosts have a higher probability of developing disseminated tuberculosis - this was particularly relevant at the dawn of HIV, prior to the availability of effective antiretroviral therapy, and more recently with the growing use of biologic agents, such as tumor necrosis factor-alpha inhibitors (etanercept, infliximab or adalimumab). Rarely disseminated disease can occur in immunocompetent hosts. Both disseminated and pulmonary tuberculosis have a higher incidence in men [3].

Urogenital tuberculosis is the third most common form of extrapulmonary TB [4], with an estimated incidence of 2-20% of cases. The entire male genital tract can be affected, and infection occurs via hematogenous spread from the primary disease site. The most common form is epididymitis, presenting clinically as a nodule in the scrotum or epididymal region. Tuberculosis should be suspected in the setting of relevant signs and symptoms, such as nodular scrotal, testicular, or prostatic lesions or nonhealing genital ulcers, coupled with a compatible epidemiologic setting. Diagnosis is confirmed by positive urine culture for *M. tuberculosis* or, in the absence of urinary involvement, with a positive culture and/or acid-fast stain in biopsy tissue of the affected site. Treatment should be promptly started, and if surgery is required, it should be deferred until after four weeks of tuberculostatic medications [4].

Central nervous system forms of the disease affect an estimated 1.5% of patients with tuberculosis and include tuberculous meningitis, tuberculoma, and spinal arachnoiditis, with meningitis being the predominant form [5]. It has a high mortality and complication rate and so treatment with tuberculostatics and glucocorticoids should be promptly started upon suspicion. The most typical presentation is similar to bacterial meningitis, with symptoms including headache, fever, and vomiting. Tuberculous meningitis can also present in a less typical way, with altered mental status, personality changes, coma, and cranial nerve palsies (mostly cranial nerves II and VI). The disease evolves in three phases: 1) early prodromal phase, with a low-grade fever and headache but no focal neurologic signs; 2) meningitic phase, with more evident neurologic signs, that can range from meningismus, lethargy, and confusion to cranial nerve palsies or hemiparesis; and 3) paralytic phase, in which the patient is in a stupor or comatose state or can present with seizures and hemiparesis. Diagnosis should be suspected in the setting of compatible clinical manifestations and a relevant epidemiologic history; cerebrospinal fluid (CSF) analysis typically shows a lymphocytic pleocytosis with elevated protein and low glucose concentrations. Definite diagnosis requires the identification of acid-fast bacilli, a positive CSF culture for *M. tuberculosis,* or a positive polymerase chain reaction (PCR) test. However, treatment should not be delayed until diagnosis confirmation and can even be started before lumbar puncture, as the acid-fast smear and culture remain positive for a few days and the PCR up to one month after treatment initiation. Mortality rates in tuberculous meningitis range from 55 to 75% [6,7].

## Case presentation

A 72-year-old male presented to the emergency department (ER) with sudden onset right hemiparesis and aphasia. He had a history of hypertension, hypercholesterolemia, and depression and was medicated with indapamide, simvastatin, paroxetine, and trazodone. In the previous two months, he had lost 17 kg and started having trouble swallowing.

On presentation, the patient was emaciated. He was afebrile, normotensive, and normocardic; lung and heart auscultation presented no relevant findings. He had homonymous right hemiparesis with ipsilateral facial paresis, conjugated eye deviation to the right with rhythmic horizontal nystagmus, and a right Babinski sign. Blood count presented mild anemia (hemoglobin of 11.5 g/dL) andlymphopenia (0.35x10^3^/uL); biochemistry showed a slight elevation in C-reactive protein (1.27 mg/dL). CT and angioCT showed no signs of stroke or mass. Diazepam was given to rule out seizures. While under observation, 4-6 hours after admission, limb paresis improved, but facial paresis persisted, which seemed consistent with a seizure. The patient was also more alert and able to speak, despite maintaining a slight speech impairment caused by sialorrhea and partial tongue-based obstruction.

Approximately 12 hours after admission, the patient developed a fever. He was started on ceftriaxone and ampicillin once blood and CSF were collected for cultures. CSF was slightly cloudy but colorless and watery; chemistry showed 203 leukocytes/uL (79% polymorphonuclears), elevated proteins (139.2 mg/dL, with 90.1 mg/dL of albumin), and discrete glycose consumption (32 mg/dL).

Two months prior to this admission, the patient presented to the emergency room complaining of a testicular mass. Echography suggested the presence of an abscess, with the need for urgent orchidectomy. He was submitted to the surgical procedure in a different hospital. Medical records were obtained, and the surgical specimen pathology was reviewed. Histology showed total disruption of the normal testicular architecture, with multiple and extensive areas of necrotizing granulomatous inflammation, with granulomas composed of epithelioid histiocytes and necrotic and abscessed areas. Gram, periodic-acid Schiff, Grocott, and Ziehl-Neelsen staining were negative for bacteria, fungi, or acid-alcohol-resistant bacilli. A molecular biology PCR test was positive for the *Mycobacterium tuberculosis* complex, confirming the diagnosis of genitourinary tuberculosis involving the left testicle, epididymis, vas deferens, and spermatic cord.

In view of these findings, the patient was started on corticosteroids, isoniazid, rifampin, pyrazinamide, and ethambutol; previously started antibiotics were not stopped, pending microbiology results. A PCR of *M. tuberculosis* complex was requested on CSF, which was positive; cultures of CSF were also positive for the same pathogen. Despite adequate treatment, the patient's health status deteriorated further, and he died on the third day of hospitalization.

Retrospectively, we concluded that surgery histology and molecular results were validated by the pathologist, but diagnosis notification was not reported to the national epidemiologic vigilance system (Sistema Nacional de Vigilância Epidemiológica - SINAVE), and information was not sent to the surgeon who operated and followed up on the patient. The surgeon also didn't review the histology results upon the post-surgery appointment. Hence, genitourinary tuberculosis was not promptly diagnosed, and the patient wasn't started on tuberculostatic drugs.

## Discussion

The number of new cases of tuberculosis in Portugal has been slowly diminishing, and in 2015 it became officially a low-incidence country (defined as 20 or fewer new cases per 100,000 habitants per year) [8]. Nevertheless, Portugal remains the Western Europe country with the highest incidence of tuberculosis. Despite the existence of notification systems and a legal obligation for clinicians to notify new cases, some studies estimate that the real incidence could be up to 30% superior to the official number [9]. In 2020, 1465 new diagnoses were reported, most of which were cases of pulmonary tuberculosis. Only 78 were cases of severe tuberculosis (disseminated, meningeal, or of the central nervous system), comprising 5.3% of the total reported cases [8].

Our patient presented no known risk factors for disseminated tuberculosis other than being an elderly male from a previously high-incidence country [10] - he was not immunocompromised (HIV was negative, and he was not a diabetic) nor under immunosuppressants and had no significant comorbidities that could weaken his immune response. He probably had contact with the disease during the course of his life and could have latent tuberculosis. Dwindling of cell-mediated immunity associated with age [1] could have caused a shift in the quiescent state of the bacilli, leading to genitourinary disease. Had the pathologic information been relayed to the appropriate parties, treatment could have been initiated two months earlier, potentially saving his life.

Medical error is defined as a preventable adverse effect of medical care. Most errors are caused by faulty systems, processes, and conditions that lead people to make mistakes or fail to prevent them [11]. A systematic review and meta-analysis concluded that the prevalence of preventable patient harm could be as high as 6%, with 12% of those errors being severe or fatal [12]. It is safe to assume that human error is not eradicable, and as such, the more we know about possible events, the better we can prevent them. Institutions should create systems that facilitate error reporting, not for assigning responsibility, but to develop prevention and monitoring strategies. The development of shared databases of medical errors [13] would facilitate widespread learning from a single event and the implementation of prevention strategies. Notification systems such as SINAVE are important for individuals as well as for public health motives, and studies consider them particularly efficient in Portugal [9].

## Conclusions

In Portugal, some labs generate automatic alerts for specific transmittable diseases (such as tuberculosis or carbapenemase-producing *Klebsiellas*), but others rely on clinicians consulting the results for the tests on a shared platform. The absence of systematic alerts increases the likelihood of missed results and delayed diagnosis.

In the present case, failure to review the pathology report after surgery, coupled with the absence of notification from the laboratory, resulted in a late diagnosis of a treatable disease and contributed to the death of this patient. We firmly believe that automated notification systems should be universal as a safety measure to minimize human error. We also defend that medical error should be openly discussed and that institutions should promote its report and debate as a way to identify systemic flaws and promote individual and institutional growth.
